# Assessment of human health risk induced by 7 metals exposure through drinking water in Urumqi, Xinjiang, China

**DOI:** 10.1097/MD.0000000000039864

**Published:** 2024-09-27

**Authors:** Jie Li, Guanxin Ding, Qin Lin

**Affiliations:** a Center for Environmental Health and Endemic Disease Control, Xinjiang Uygur Autonomous Region Center for Disease Control and Prevention, Urumqi, Xinjiang, China; b Yantai City Center for Disease Control and Prevention, Yantai, Shandong, China.

**Keywords:** drinking water, health risk assessment, metals

## Abstract

Heavy metal water pollution refers to the abnormal concentration of metal elements and their compounds in water with a relative density of >4.5, which causes the water quality to decline or deteriorate. To assess the presence of 7 metals in drinking water in a city in Xinjiang and the health risks to the human body caused by drinking the water and to provide a scientific basis for health risk management for drinking water. In 2021, 114 monitoring points were set up in Urumqi, Xinjiang, China, and 228 water samples were collected in the dry and in the wet seasons to monitor water quality. Using the *Standards for drinking water quality* (GB 5749-2006), the concentrations of 7 metals were measured, and a method recommended by the United States Environmental Protection Agency was used to assess health risks. A total of 228 water samples were collected and measured, and 227 met the standard, for a compliance rate of 99.56%. Except for Mn, the compliance rates for the other 6 metals were 100%. Based on noncarcinogenic health risk, the order of the 7 metals was Al > Fe > Gu > Mn > Hg > Zn > Pb, and the hazard index was 3.33 × 10^‐7^ < 1. The total noncarcinogenic health risk of 7 metals was <1, that is, within the acceptable range. Al has the highest noncarcinogenic health risk, followed by Fe.

## 1. Introduction

With social and economic development, increasing metal exposure events have occurred, with drinking water being polluted to varying degrees. Air, water, and soil contamination are the basis of many environmental problems. Fresh water is the most important natural resource on Earth, a prerequisite for life and all life forms, and a fundamental requirement for ecological diversity and sustainable development.^[1]^

Drinking water is the basic need for human survival, and the health and safety of drinking water is related to the health of the masses, and it is a major public health safety issue. Heavy metals are toxic to the nervous, digestive and reproductive systems of the human body and are environmental pollutants.^[2,3]^ Drinking water is an important means through which humans ingest heavy metals, and drinking water contaminated with heavy metals can potentially harm human health.^[4]^ Heavy metals cannot be decomposed in water, and have the characteristics of high toxicity, long persistence, difficult degradation, etc, and are often detected in water bodies. According to the survey of the World Health Organization, 89% of the population to tap water for drinking water,^[5]^ water source pollution caused by direct or indirect harm to human health is a long, subtle process, so long-term drinking water containing high concentrations of heavy metals, metallike, and organic pollutants will have a certain impact on human health.^[6]^

If people living in drinking water polluted areas are exposed to or ingested excessive chromium, they are prone to rhinitis, tuberculosis and other diseases. The harm of lead pollution to the human body is mainly shown as affecting human intellectual development and skeletal development, causing dyspepsia and endocrine disorders, leading to anemia, hypertension and arrhythmia, and destroying kidney function and immune function.^[7]^ Excessive manganese causes damage to human nervous, reproductive, respiratory, and other systems.^[8]^ Excess iron can lead to liver, kidney, tumors and other diseases.^[9]^ Exposure to methylmercury causes a severe neurodegenerative condition known as Minamata disease. In addition, mercury is responsible to produce free radicals leading to DNA damage.^[10]^

During the production process of industrial enterprises, pollutants containing heavy metals are imported into surface water through various ways such as efflux production wastewater and exhaust gas discharge, causing water pollution and threatening people’s health and safety, especially those who are exposed to the surrounding enterprises for a long time.

Urumqi, the capital of Xinjiang Uygur Autonomous Region, is a transportation and communication hub connecting the north and south of Xinjiang and connecting the mainland of China with Central and Western Asia and Europe. The second Eurasian land bridge is the bridgehead in western China and an important gateway to China’s opening to the west. It is the 16th emerging city in Chinese Mainland in terms of comprehensive strength, and also the largest city in the 5 Central Asian countries. In recent years, the urbanization process of Urumqi has accelerated,^[11]^ and both the population and economy have developed rapidly. At the same time, there are also some industrial enterprises that discharge wastewater that does not meet the standards, which is illegally discharged or leaked.^[12]^ There are certain drinking water safety issues in all counties and cities under the jurisdiction.

Therefore, this study investigated and analyzed metals and heavy metals in drinking water of Umuqi City in 2021, and used the health risk assessment model recommended by USEPA to assess its health risks, so as to understand the status of metals and heavy metals in drinking water of Urumqi and provide scientific basis for the construction of national water reform project and the prevention of heavy metal poisoning.

## 2. Subjects and methods

### 2.1. Research subjects

**Figure 1.**
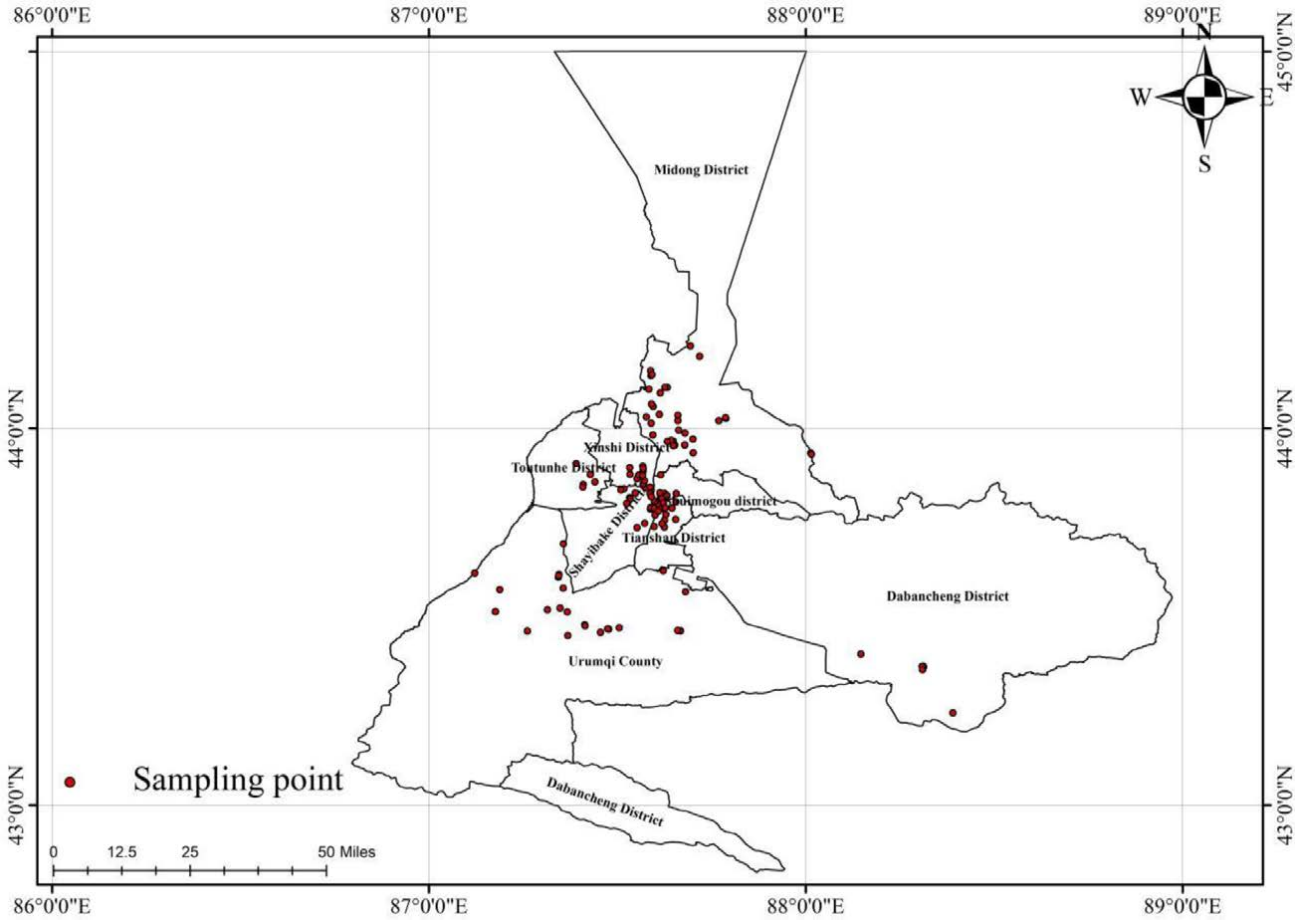
Geographical location and sampling points of Urumqi City.

In 2021, there were the 48 centralized water supply projects in a city in Xinjiang. Within the scope of the 48 centralized water supply projects, 114 monitoring points were set up, and 1 water sample was collected during the dry season (March–May) and during the wet season (July–August) for monitoring. A total of 228 water samples were collected throughout the year, including 50 finished water samples, 28 secondary water supply samples, and 150 tap water samples (Fig. 1).

### 2.2. Detection method

Referencing the *Standard examination methods for drinking water* (GB/T 5750-2006),^[13]^ water samples were collected, stored, transported, and tested. When the test result was lower than the method detection limit, half the method detection limit was used.

### 2.3. Assessment method

The detection results for 7 metals, that is, Hg, Pb, Fe, Mn, Zn, Gu, and Al, in water samples were assessed using the *Standards for Drinking Water Quality* (GB/T 5749-2006).^[14]^ For the rural centralized water supply project, with a daily water supply below 1000 m^3^ (or a population below 10,000), the limits for Fe and Mn are 0.5 mg/L and 0.3 mg/L, respectively.

### 2.4. Health risk assessment method

The method recommended by the United States Environmental Protection Agency (US EPA) was used to assess the health risk of 7 metals in 228 water samples. The assessment method includes 4 steps: hazard identification, dose–response relationship assessment, exposure assessment, and risk characterization.^[15,16]^ To account for the different characteristics of toxic substances in the body, 2 models (carcinogenic and noncarcinogenic risk assessments) were used.^[17,18]^

### 2.5. Hazard identification

Using the results of human carcinogenicity studies and animal carcinogenicity experimental data, the International Agency for Research on Cancer divides chemicals into 4 groups. In this study, Pb, Hg, Fe, Mn, Zn, Gu, and Al were classified as noncarcinogens in the risk assessment.

### 2.6. Dose–response relationship assessment

Using the reference doses (RfDs) for noncarcinogenic chemicals published by the US EPA,^[19,20]^ RfDs of Pb, Hg, Gu, Fe, Mn, Zn, and Al were 1.4 × 10^‐3^, 3 × 10^‐4^, 3.7 × 10^‐2^, 0.7, 0.14, 0.3, and 1 mg/(kg·d), respectively.

### 2.7. Exposure assessment

The health risk of oral exposure to drinking water was calculated using the noncarcinogen risk assessment model. The relevant parameters of the population were obtained from the *Chinese Exposure Factors Handbook* (Adults) for the Xinjiang Uygur Autonomous Region.^[21]^

The noncarcinogenic risk assessment model was $R_{i}^{n}=\left(Di\times 10^{-6}/RfDi\right)/L$, and the mean daily exposure of pollutants through drinking water was calculated as $D_{i}=Ci\times IR\times EF\times ED/\left(BW\times AT\right)$. In the model, $R_{i}^{n}$ is the mean personal health risk of noncarcinogenic substance i ingested through drinking water, a^-1^; $RfD_{i}$ is the RfD of noncarcinogenic substance i ingested through drinking water, mg/(kg·d); L is the mean life expectancy, year; $C_{i}$ is the mass concentration of noncarcinogenic substances, mg/L; IR is the mean daily water intake of adults, L/d; BW is the mean adult body weight, kg. The parameters in the models were determined based on the situation in Xinjiang Uygur Autonomous Region, that is, IR, 1.975 L/d; BW, 64.2 kg;^[17]^ exposure frequency, 365 days/year; continuous exposure time, 74.18 years.^[22]^ Mean exposure time = continuous exposure time × exposure frequency. The values for RfD and safety factor indicators in the assessment model are based on US EPA standards.^[23]^

### 2.8. Risk characterization

The hazard factors $R_{i}^{C}$ of various noncarcinogens were summed to obtain the hazard index: $HI=\overset{k}{\underset{i=1}{\sum }}\;R_{i}^{n}$.

### 2.9. Health risk assessment criteria

The health risks of 7 metals and heavy metals were determined using the assessment criteria of the EPA. A hazard quotient <1 was used as the standard for the risk assessment of noncarcinogens.^[24,25]^

### 2.10. Statistical analysis

Excel 2012 was used for data processing, and SPSS 24.0 was used for statistical analysis. The compliance rate was calculated. Comparisons between groups were carried out using Pearson χ^2^ test or Fisher exact test, and count data are presented as frequencies. Comparisons between groups that did not conform to a normal distribution were performed using a nonparametric test for 2 or more independent samples (Mann–Whitney U test or Kruskal–Wallis H test). Medians are used to describe the concentrations of the 7 indicators. All statistical analyses were two-sided tests, and *P* < .05 indicated that a difference was statistically significant.

## 3. Results

**Figure 2.**
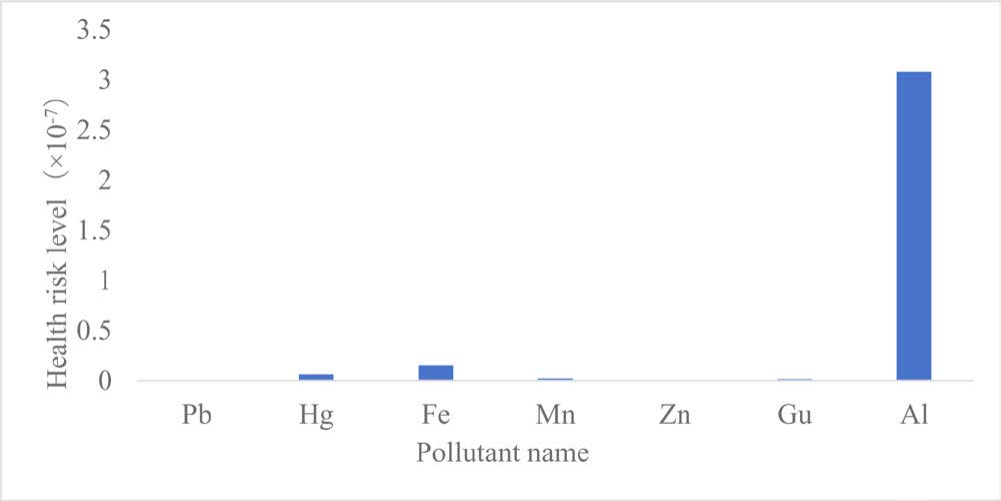
Concentrations and health risk levels of 7 metals in drinking water in a city in Xinjiang in 2021.

Concentrations and health risk levels of 7 metals and heavy metals in drinking water A total of 228 water samples were collected in a city in Xinjiang in 2021; 227 met the standard, for a compliance rate of 99.56%. Except for Mn, the compliance rates for the other 6 metals were 100%. The order of the 7 metals based on noncarcinogenic health risk was Al > Fe > Gu > Mn > Hg > Zn > Pb, and the hazard index was 3.33 × 10^‐7^ < 1 (Table 1 and Fig. 2).

**Table 1:** Concentrations and health risk levels of 7 metals in drinking water in a city in Xinjiang in 2021.

Indicator	Number of samples that met the standard	Compliance rate (%)	Concentration (10^-3^)/(mg/L)		Health risk level (M)
			Range	M (P25, P75)	
Pb	228	100.00	0.14–4.00	1.25 (0.25, 1.25)	1.28 × 10^‐10^
Hg	228	100.00	0.05–0.25	0.06 (0.05, 0.14)	6.15 × 10^‐9^
Fe	228	100.00	4.00–241.00	150.00 (25.00, 150.00)	1.54 × 10^‐8^
Mn	227	99.56	1.00–30.00	10.00 (10.00, 10.00)	2.20 × 10^‐9^
Zn	228	100.00	1.00–640.00	3.75 (3.25, 25)	3.85 × 10^‐10^
Gu	228	100.00	2.00	2.00 (2.00, 2.00)	1.54 × 10^‐9^
Al	228	100.00	1.25–90.00	4.00 (4.00, 9.00)	3.08 × 10^‐7^

**Figure 3.**
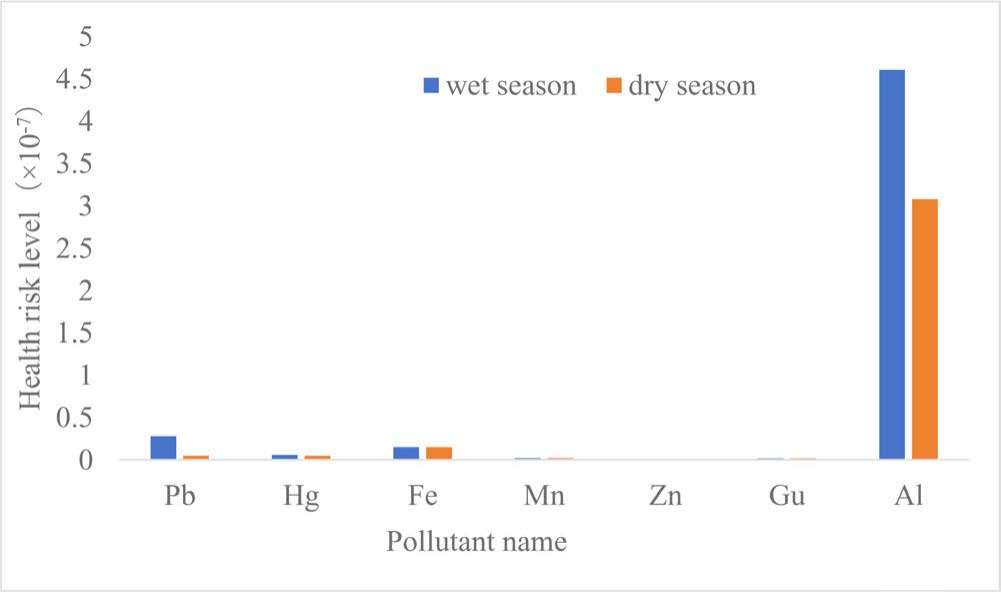
Concentrations and health risk levels of 7 metals in drinking water during different seasons in a city in Xinjiang (2021).

Concentrations and health risk levels of 7 metals and heavy metals in drinking water in different seasons Between different seasons in 2021, there was no significant difference in the compliance rates for 7 metals in drinking water in a city in Xinjiang (*P* > .05). The total noncarcinogenic risk of 7 metals and heavy metals ingested by residents in a city in Xinjiang was 5.14 × 10^‐7^ and 3.38 × 10^‐7^ in the wet season and dry season, respectively. The concentrations and health risk levels of Hg, Al and Fe in the wet season were significantly higher than those in the dry season (Mann–Whitney U test, *P* < .01). The concentrations and health risk levels of Pb, Gu, Mn, and Zn were not significantly different between the wet season and dry season (*P* > .05) (Table 2 and Fig. 3).

**Table 2:** Concentrations and health risk levels of 7 metals in drinking water during different seasons in a city in Xinjiang (2021).

Indicator	Season	Compliance rate (%)	Concentration (10^-3^)/(mg/L)		*Z*	*P*	Health risk level (M)	*Z*	*P*
			Range	M (P25, P75)					
Pb	Wet season	100	0.14–4.00	1.25 (0.25, 1.25)	*‐*0.056	.956	2.75 × 10^-8^	‐0.044	.965
	Dry season	100	0.14–1.25	0.25 (0.25, 1.25)			5.49 × 10^-9^		
Hg	Wet season	100	0.05–0.25	0.06 (0.05, 0.06)	-3.528	.000	6.15 × 10^-9^	-3.663	.000
	Dry season	100	0.05–0.25	0.05 (0.05, 0.05)			5.13 × 10^-9^		
Fe	Wet season	100	4.00–241.00	150.00 (47.50, 150.00)	-3.094	.002	1.54 × 10^-8^	-3.232	.001
	Dry season	100	4.00–150.00	150.00 (25.00, 150.00)			1.54 × 10^-8^		
Mn	Wet season	99.12	1.00–30.00	10.00 (10.00, 10.00)	-0.304	.761	2.20 × 10^-9^	-0.482	.630
	Dry season	100	1.00–25.00	10.00 (10.00, 20.00)			2.20 × 10^-9^		
Zn	Wet season	100	1.00–350.00	3.75 (3.75, 25)	-0.158	.875	3.85 × 10^-10^	-0.050	.960
	Dry season	100	1.50–640.00	3.75 (3.25, 25.00)			3.85 × 10^-10^		
Gu	Wet season	100	2	2.00 (2.00, 2.00)	0.000	1.000	1.54 × 10^-9^	0.000	1.000
	Dry season	100	2	2.00 (2.00, 2.00)			1.54 × 10^-9^		
Al	Wet season	100	1.25–90.00	6.00 (4.00, 50.00)	-7.533	.000	4.61 × 10^‐7^	-7.533	.000
	Dry season	100	4.00–20.00	4.00 (4.00, 4.00)			3.08 × 10^-7^		

*Note*: There were 114 water samples for the dry season and for the wet season.

**Figure 4.**
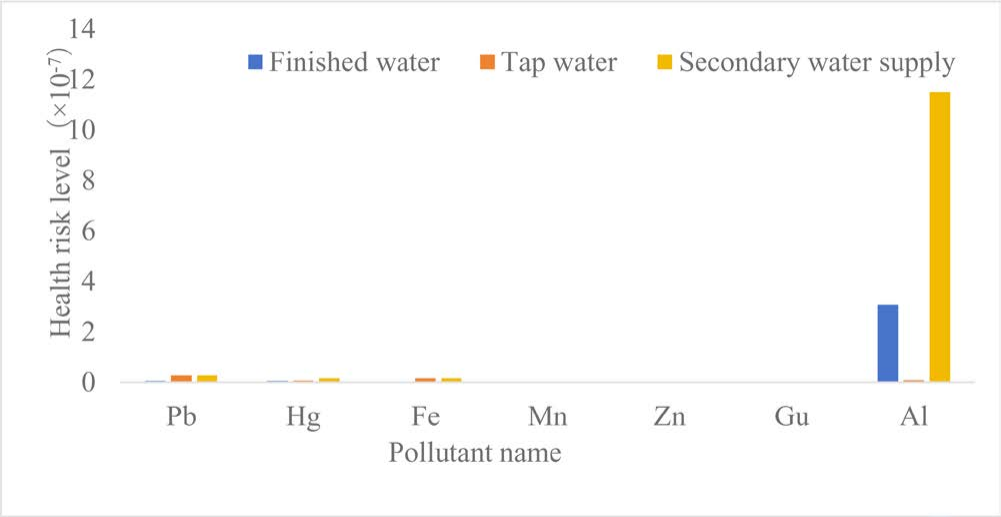
Concentrations and health risk levels of 7 metals in drinking water from different water sample types in a city in Xinjiang (2021).

Concentrations and health risk levels of 7 metals and heavy metals in different types of drinking water In 2021, the compliance rates for 7 metals in drinking water in a city in Xinjiang were not significantly different between different water sample types (*P* > .05). The total noncarcinogenic risk associated with residents in a city in Xinjiang drinking finished water, tap water and secondary water was 3.24 × 10^‐7^, 3.87 × 10^‐7^, and 1.26 × 10^‐6^, respectively. The concentrations and health risk levels of Pb, Hg, Al, and Fe were significantly higher in secondary water than in finished water and tap water (secondary water supply > finished water > tap water) (Kruskal–Wallis test, *P* < .05). The concentrations and the health risk levels of Gu, Mn, and Zn were not significantly different between the wet season and dry season (*P* > .05) (Table 3 and Fig. 4).

**Table 3:** Concentrations and health risk levels of 7 metals in drinking water from different water sample types in a city in Xinjiang (2021).

Indicator	Type of water sample	Compliance rate (%)	Concentration (10^‐3^)/(mg/L)		χ^*2*^	*P*	Health risk level (M)	χ^*2*^	*P*
			Range	M (P25, P75)					
Pb	Finished water	100	0.14–4.00	0.25 (0.14, 0.25)	39.287	.000	5.49 × 10^-9^	37.443	.000
	Tap water	100	0.14–1.25	1.25 (0.25, 1.25)			2.75 × 10^-8^		
	Secondary water supply	100	1.25	1.25 (1.25, 1.25)			2.75 × 10^-8^		
Hg	Finished water	100	0.05–0.25	0.05 (0.05, 0.05)	34.510	.000	5.13 × 10^-9^	34.754	.000
	Tap water	100	0.05–0.25	0.06 (0.05, 0.15)			6.15 × 10^-9^		
	Secondary water supply	100	0.06–0.25	0.15 (0.06, 0.25)			1.54 × 10^-8^		
Fe	Finished water	100	4.00–150.00	25.00 (25.00, 61.00)	41.667	.000	2.56 × 10^-9^	38.082	.000
	Tap water	100	4.00–241.00	150.00 (25.00, 150.00)			1.54 × 10^-8^		
	Secondary water supply	100	150.00–234.00	150.00 (150.00, 150.00)			1.54 × 10^-8^		
Mn	Finished water	98	2.00–25.00	2.00 (2.00, 25.00)	0.117	.943	4.39 × 10^-10^	0.110	.947
	Tap water	100	1.00–30.00	10.00 (2.00, 10.00)			2.20 × 10^-9^		
	Secondary water supply	100	1.00–20.00	10.00 (10.00, 10.00)			2.20 × 10^-9^		
Zn	Finished water	100	1.00–25.00	3.75 (1.50, 25.00)	1.062	.588	3.85 × 10^-10^	0.984	.612
	Tap water	100	1.00–640.00	3.75 (3.75, 25.00)			3.85 × 10^-10^		
	Secondary water supply	100	3.75–340.00	3.75 (3.75, 3.75)			3.85 × 10^-10^		
Gu	Finished water	100	2	2.00 (2.00, 2.00)	0.000	1.000	1.54 × 10^-9^	0.000	1.000
	Tap water	100	2	2.00 (2.00, 2.00)			1.54 × 10^-9^		
	Secondary water supply	100	2	2.00 (2.00, 2.00)			1.54 × 10^-9^		
Al	Finished water	100	4.00–90.00	4.00 (4.00, 4.00)	7.988	.018	3.08 × 10^-7^	7.988	.018
	Tap water	100	1.25–70.00	4.00 (4.00, 30.00)			3.08 × 10^-7^		
	Secondary water supply	100	1.25–70.00	15.00 (4.00, 50.00)			1.15 × 10^-6^		

*Note*: There were 50, 150, and 28 samples of finished water, tap water, and secondary water, respectively.

**Figure 5.**
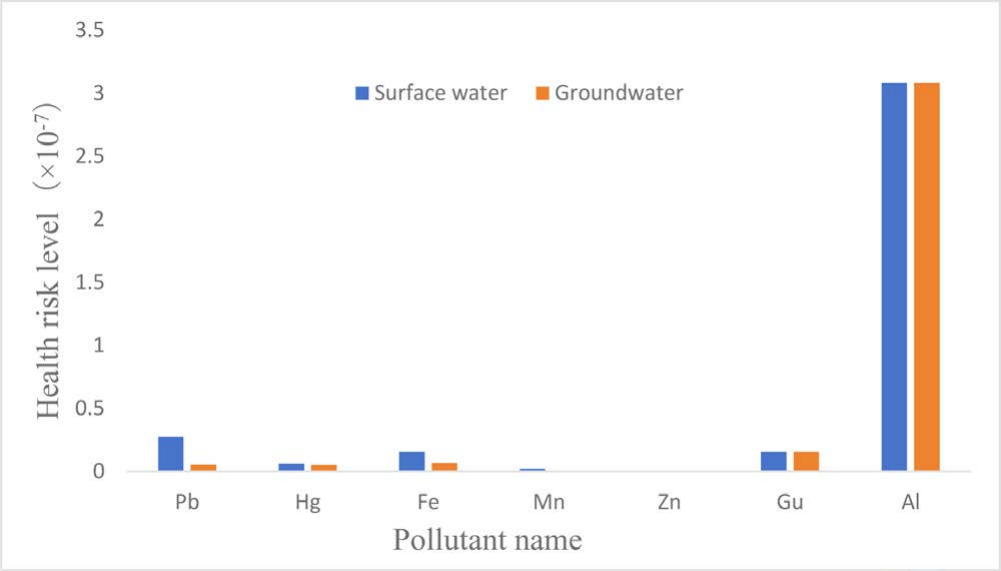
Concentrations and health risk levels of 7 metals in drinking water from different water source types in a city in Xinjiang (2021).

Concentrations and health risk levels of 7 metals and heavy metals in drinking water from different sources In 2021, the compliance rates for 7 metals in drinking water in a city in Xinjiang were not significantly different between different water source types (*P* > .05). The total noncarcinogenic risk of surface water and groundwater as sources of drinking water for residents in a city in Xinjiang was 3.61 × 10^‐7^ and 3.28 × 10^‐7^, respectively. The concentrations and health risk levels of Pb, Hg, Fe, Mn, Al, Zn in surface water were significantly higher than those in groundwater (Mann–Whitney U test, *P* < .05). There was no significant difference in the concentration and health risk level of Gu between the wet season and dry season (*P* > .05) (Table 4 and Fig. 5).

**Table 4:** Concentrations and health risk levels of 7 metals in drinking water from different water source types in a city in Xinjiang (2021).

Indicator	Type of water source	Compliance rate (%)	Concentration (10^-3^)/(mg/L)		*Z*	*P*	Health risk level (M)	*Z*	*P*
			Range	M (P25, P75)					
Pb	Surface water	100.00	0.14–4.00	1.25 (0.25, 1.25)	-4.500	.000	2.75 × 10^-8^	-4.363	.000
	Groundwater	100.00	0.14–1.25	0.25 (0.25, 0.25)			5.49 × 10^-9^		
Hg	Surface water	100.00	0.05–0.25	0.06 (0.05, 0.25)	-2.388	.017	6.15 × 10^-9^	-2.319	.020
	Groundwater	100.00	0.05–0.25	0.05 (0.05, 0.06)			5.13 × 10^-9^		
Fe	Surface water	100.00	4.00–241.00	150.00 (25.00, 150.00)	-2.920	.003	1.54 × 10^-8^	-2.721	.007
	Groundwater	100.00	4.00–239.00	64.50 (25.00, 150.00)			6.61 × 10^-9^		
Mn	Surface water	99.29	1.00–130.00	10.00 (10.00, 25.00)	-6.887	.000	2.20 × 10^-9^	-6.571	.000
	Groundwater	100.00	2.00–25.00	2.00 (2.00, 10.00)			4.39 × 10^-10^		
Zn	Surface water	100.00	1.00–640.00	3.75 (3.75, 25.00)	-6.950	.000	3.85 × 10^-10^	-6.866	.000
	Groundwater	100.00	1.00–340.00	3.25 (1.50, 3.75)			3.33 × 10^-10^		
Gu	Surface water	100.00	2	2.00 (2.00, 2.00)	0.000	1.000	1.54 × 10^-9^	0.000	1.000
	Groundwater	100.00	2	2.00 (2.00, 2.00)			1.54 × 10^-9^		
Al	Surface water	100.00	1.25–100.00	4.00 (4.00, 30.00)	-2.309	.021	3.08 × 10^-7^	-2.309	.021
	Groundwater	100.00	4.00–70.00	4.00 (4.00, 4.00)			3.08 × 10^-7^		

*Note*: There were 141 and 87 surface water and groundwater samples, respectively.

## 4. Discussion

Heavy metal pollution in drinking water is one of the major public health problems, and the state and governments at all levels have been committed to solving and ensuring the health and safety of drinking water. Due to the low content of heavy metals and metal-like substances in drinking water, the chronic effects of long-term low-dose exposure have a greater impact on population health than the acute effects of short-term high concentration exposure. *Basic results*: The results of this study indicated that in 2021, the concentrations of 6 metals and heavy metals, that is, Pb, Hg, Gu, Fe, Zn, and Al, in drinking water in a city in Xinjiang met the *Standards for drinking water quality* (GB5749-2006), for a compliance rate of 100%; the concentration of Mn exceeded the standard.^[13]^ The total noncarcinogenic risk of 7 metals and heavy metals was <1, within the acceptable range. Al had the highest noncarcinogenic health risk, followed by Fe, suggesting that a city in Xinjiang should pay attention to the potential health hazards of Al and Fe to the population in the prevention and control of drinking water pollution and strengthen the monitoring and assessment of drinking water.

*Cause analysis of excessive Mn*: In the water samples analyzed in this paper, the concentration of Mn in one water sample of finished water from a reservoir during the wet season exceeded the standard. The water quality monitoring results for the finished water from the reservoir in the past 5 years showed that the concentration of Mn in the finished water exceeded the standard during the wet season each year but met the standard during the dry season, that is, the Mn concentration during the wet season was higher than that during the dry season, a finding that is consistent with the results of other studies.^[26,27]^ We preliminarily propose that the excessive Mn concentration is seasonal, mainly related to the water temperature, pH, and dissolved oxygen content in the reservoir. Under normal circumstances, the reservoir water is neutral (pH), and the oxygen content should maintain the Mn in the water body as slightly soluble oxides and hydroxides (solids). When the temperature is high in the wet season, the water temperature stratification in the reservoir water is more substantial and produces a density barrier effect. The upper and lower water bodies are separated by a thermocline, and the convective movement slows; therefore, the oxygen in the upper water body does not readily enter the lower water body and the bottom through the thermocline, the dissolved oxygen at the bottom is consumed by reducing pollutants and the decomposition of organic matter, and the dissolved oxygen content and pH value in the bottom layer decrease, resulting in a reduction in n-tetravalent Mn in the bottom sediment to soluble n-divalent Mn ions, thus increasing the Mn concentration in the water.

*Health risk assessment*: Health risk assessments link environmental pollution with human health and quantitatively describe the harm caused by pollution to human health.^[28–30]^ Some foreign scholars mentioned in a study conducted in Iran’s Hamidan province that exposure levels of heavy metals, especially lead exposure levels, may lead to increased incidence of colorectal cancerm.^[31]^ Studies have also reported that long-term exposure to lead can cause anemia and high blood pressure, especially in middle-aged and elderly people, however, water with a lead concentration lower than 0.05 mg/L has certain damage to the development of brain tissue in young children.^[32,33]^ In this study, the total health risk of 7 metals and heavy metals in a city in Xinjiang was on the order of 10^‐7^, which is far lower than the maximum acceptable risk recommended by the US EPA and ICRP (1 × 10^‐4^/a and 5 × 10^‐5^/a) but higher than the negligible risk levels (1 × 10^‐7^/a and 1 × 10^‐8^/a) recommended by the Royal Society of England and the Netherlands Construction and Environment Department,^[34]^ indicating that the health hazards to humans of 7 metals and heavy metals in the drinking water in a city in Xinjiang are within the acceptable range but still pose some health risks to residents. It is recommended to continue to strengthen the monitoring of metals and heavy metal indicators in drinking water in the future and to carry out health risk assessments on a regular basis to provide a theoretical basis for prioritizing the treatment of polluted drinking water. The concentrations and health risk levels of Hg, Al, and Fe in drinking water are different in different seasons, indicating that the amount of water has an impact on the concentrations and health risk levels of the above 2 metals and metalloid. It is recommended that water plants improve regulation structures, improve the water treatment process, and maintain water quality at a relatively stable level. The concentrations and health risk levels of Pb, Hg, Al, and Fe are significantly different among finished water, tap water, and secondary water, indicating that distribution links have impacts on water quality, a result that is inconsistent with findings for Shaanxi Province.^[16]^ The water transmission and distribution pipeline network in a city in Xinjiang is relatively long, residential water consumption during the wet season is high, and the pipeline network large is large; therefore, the concentrations of metals in the water are prone to change, indicating that the maintenance and management of the transmission and distribution network should be to strengthened to ensure the safety and stability of drinking water quality. The concentrations and health risk levels of Pb, Hg, Fe, Mn, Al, and Zn in surface water were higher than those in groundwater, a finding that may be related to the fact that surface water is susceptible to direct and indirect pollution by human activities, suggesting that it is necessary to strengthen the protection of surface water as a source of drinking water, to designate water source protection zones, and to regularly carry out quality monitoring of water sources to ensure the safety of drinking water for residents.

In this study, the health risk assessment model was used for the first time to assess the exposure status of 7 metals and heavy metals in drinking water of Urumqi, Xinjiang. The concentration of the 7 environmental pollutants was not only evaluated, but also the physiological characteristics of the pollutants such as toxicological properties and body weight were taken into account. The final calculated health risk results can not only explain the concentration of pollution, but also more directly reflect the threat to human health.

## 5. Conclusion

The total noncarcinogenic health risk of 7 metals was <1, that is, within the acceptable range. Al has the highest noncarcinogenic health risk, followed by Fe, suggesting that Urumqi should pay attention to the potential health hazards of Al and Fe for the prevention and control of drinking water pollution and strengthen drinking water monitoring and assessments.

## 6. Limitation(s)

This study only analyzed the health risks of metal exposure through drinking water and did not address the risks of other exposure routes to the human body.

This study only considered the route of exposure to drinking water, did not address the risk after entering the human body, and did not consider the impact of different ethnic groups, age, sex, activity intensity, and drinking habits in the health risk assessment. Therefore, this study only serves as a preliminary exploration of the health risk assessment of metals and heavy metals in drinking water in a city in Xinjiang. Future studies should fully quantify the effects of contaminant exposure in drinking water on human health.

In the future, we should continue to strengthen and improve the research in this area, especially the research on the risk threshold, in order to comprehensively quantify the impact of exposure to pollutants in drinking water on human health.

## Acknowledgments

We acknowledge the efforts of all coauthors, editor, and reviewers for improving this manuscript.

## Author contributions

**Data curation:** Jie Li.

**Formal analysis:** Qin Lin.

**Funding acquisition:** Jie Li.

**Investigation:** Qin Lin.

**Methodology:** Guanxin Ding.

**Software:** Guanxin Ding.

**Writing – original draft:** Jie Li, Guanxin Ding.

**Writing – review & editing:** Guanxin Ding, Qin Lin.
